# The Identification of Human Translational Biomarkers of Neuropathic Pain and Cross-Species Validation Using an Animal Model

**DOI:** 10.1007/s12035-022-03124-7

**Published:** 2022-11-24

**Authors:** Bethan Young, John Stephenson, Barira Islam, Nikita N. Burke, Elaine M. Jennings, David P. Finn, Patrick C. McHugh

**Affiliations:** 1 Centre for Biomarker Research, University of Huddersfield, Huddersfield, HD1 3DH UK; 2 Department of Pharmacy, School of Applied Sciences, University of Huddersfield, Huddersfield, HD1 3DH UK; 3 School of Human and Health Sciences, University of Huddersfield, Huddersfield, HD1 3DH UK; 4 School of Nursing and Midwifery, University of Galway, Galway, Ireland; 5 Centre for Pain Research, University of Galway, Galway, Ireland; 6 Galway Neuroscience Centre, University of Galway, Galway, Ireland; 7 Pharmacology and Therapeutics, University of Galway, Galway, Ireland

**Keywords:** Chronic neuropathic pain, *CASP9*, SNL, Biomarker, Dorsal horn, AUROC analysis

## Abstract

Neuropathic pain is a common chronic condition, which remains poorly understood. Many patients receiving treatment continue to experience severe pain, due to limited diagnostic/treatment management programmes. The development of objective clinical diagnostic/treatment strategies requires identification of robust biomarkers of neuropathic pain. To this end, we looked to identify biomarkers of chronic neuropathic pain by assessing gene expression profiles in an animal model of neuropathic pain, and differential gene expression in patients to determine the potential translatability. We demonstrated cross-species validation of several genes including those identified through bioinformatic analysis by assessing their expression in blood samples from neuropathic pain patients, according to conservative assessments of significance measured using Bonferroni-corrected *p*-values. These include *CASP5* (*p* = 0.00226), *CASP8* (*p* = 0.00587), *CASP9* (*p* = 2.09 × 10^−9^), *FPR2* (*p* = 0.00278), *SH3BGRL3* (*p* = 0.00633), and *TMEM88* (*p* = 0.00038). A ROC analysis revealed several combinations of genes to show high levels of discriminatory power in the comparison of neuropathic pain patients and control participants, of which the combination *SH3BGRL3*, *TMEM88*, and *CASP9* achieved the highest level (AUROC = 0.923). The *CASP9* gene was found to be common in five combinations of three genes revealing the highest levels of discriminatory power. In contrast, the gene combination *PLAC8*, *ROMO1*, and *A3GALT2* showed the highest levels of discriminatory power in the comparison of neuropathic pain and nociceptive pain (AUROC = 0.919), when patients were grouped by S-LANSS scores. Molecules that demonstrate an active role in neuropathic pain have the potential to be developed into a biological measure for objective diagnostic tests, or as novel drug targets for improved pain management.

**Supplementary Information:**

The online version contains supplementary material available at 10.1007/s12035-022-03124-7.

## Introduction

Neuropathic pain affects approximately 7.4 million people in the UK, and most patients receiving treatment still experience moderate-to-severe pain [1]. Patient quality of life is compromised by inability to work, reduced mobility and independence, sleep disturbances, and medication side effects that often compromise patient adherence [2]. Significant social and economic costs are associated with neuropathic pain due to lack of effective diagnostic and treatment strategies [3].

Neuropathic pain can result from damage to peripheral nerves, and manifests clinically as spontaneous pain, as painful responses to innocuous stimuli (allodynia), or as a heightened pain response (hyperalgesia) [4]. Neuropathic pain is often a consequence of traumatic nerve injury, or a result of severe chronic inflammation over time [5]. The complexity of chronic pain is further exacerbated as it is also associated with numerous peripheral and centrally mediated disorders, such as diabetes and multiple sclerosis [6], as well as the presence of multiple pain manifestations within an individual patient. These factors result in a highly complex patient profile that makes pinpointing diagnosis difficult, resulting in ineffective treatment strategies being applied. At present, neuropathic pain is clinically diagnosed using a combination of characteristic painful symptoms, or altered sensation, as well as pain questionnaires, such as the Leeds Assessment of Neuropathic Pain Signs and Symptoms (S-LANSS) pain scale [7]. The subjective nature of questionnaires, coupled with the underlying variability in the manifestation of pain, makes it difficult to accurately assess and diagnose neuropathic pain, or its subtypes. Moreover, the pain experience can vary over time, and the associated psychological distress can often distort the patient’s assessment of their pain [8]. Furthermore, clinical observation has limited implications for treatment strategy because different mechanisms may produce the same outward symptom. Variability in screening tool results has been reported [9], and 10–20% of neuropathic pain cases cannot be identified this way [10], which can lead to incorrect diagnoses and inappropriate treatment recommendations. The clear deficit in effective treatments and objective diagnostic tools therefore provides the impetus to identify and validate novel and translational diagnostic biomarkers to facilitate early intervention in neuropathic pain.

Neuropathic pain is managed with drugs available for other disorders, which demonstrate varying efficacies in pain management across different types of neuropathic pain [11]. First-line treatments include antidepressants, anticonvulsant drugs, anti-inflammatories, and topical lidocaine. Opioid analgesics are generally considered second-line due to their addictive properties and high cost [3]. Medication regimes for neuropathic pain are often developed through trial and error, as the combination of drugs with the greatest pain relief and fewest adverse side effects is determined gradually. This can be further hindered with the variability in diagnostic assessments, which delays symptom management and prolongs patient discomfort. Severe side effects may also require patients to seek additional medical assistance to manage these effects, leading to polypharmacy. Non-drug treatments, including transcutaneous electrical nerve stimulation (TENS) and acupuncture, are also available for neuropathic pain, but are supported with limited clinical evidence [12, 13]. To date, there are no definitive treatments available specifically for neuropathic pain, and the development of specific biomarkers may lead to more effective clinical management and treatment options for neuropathic pain patients.

Several studies have looked to identify potential biomarkers for predicting neuropathic pain, including studies that have demonstrated cross-species validation [14–18], although no specific biomarkers for clinical use have yet been identified. Our previous work has identified several interesting candidate molecules including cysteine-aspartic acid protease 5 (*CASP5*) [14, 15], melanocortin-1 receptor (*MC1R*), and tissue inhibitor of matrix metalloproteinase-1 (*TIMP1*) in human blood and the rat spinal nerve ligation (SNL) model [15]. In the present study, we have looked to identify additional robust biomarkers for the purpose of improved clinical diagnosis and management of neuropathic pain by exploring the transcriptomic profiles of the dorsal horn from SNL model rats 39 days after surgery and reverse-translate in blood samples from human neuropathic pain participants. The dorsal horn was chosen, as this tissue is critical in normal sensory processing and in pain pathology [19], as well as being a target for therapeutic modulation of pain-related pathways. A cross-species analysis would not only provide us with insights into the mechanism of neuropathic pain (rat model), but also how it manifests in the human condition. Using human blood allows us to (A) determine if pain molecules/pathways identified in the rat dorsal horn are reflected in the human circulatory system; studies have raised the possibility that changes in blood–brain barrier permeability occur in chronic pain [20, 21] and therefore the potential for pain-associated molecules egressing into the peripheral blood system; and (B) use a readily accessible tissue source for the development of biomarkers for clinical utility in chronic pain. Through this approach, we have identified several potential neuropathic pain biomarkers, both previously reported and newly discovered. These biomarkers may form part of a robust diagnostic profile or provide useful leads for drug targeting/repurposing, to improve the diagnosis and treatment of neuropathic pain. In addition, the work will yield a human-validated molecular profile for the preclinical development of novel or repurposed analgesic compounds.

## Methods

### Animal Husbandry, L5 SNL Surgery, and Tissue Harvest

The experimental procedures for animal husbandry, surgery, and tissue harvest were approved by the Animal Care and Research Ethics Committee, National University of Ireland, Galway, Ireland, and carried out under license from the Department of Health in the Republic of Ireland and in accordance with EU Directive 2010/63. The L5 spinal nerve ligation (L5 SNL) model of neuropathic pain in adult male Sprague Dawley rats (Harlan, UK) was implemented as described previously [22]. The rats were housed singly with free access to food and water, under a controlled temperature (21 ± 2 °C) and 12-h light–dark cycling. One week was allowed for acclimatization prior to L5 SNL (*n* = 8) or sham (*n* = 8) surgery. Euthanasia was by decapitation 39 days post-surgery and tissue was harvested from the spinal cord dorsal horn (DH) ipsilateral to the side of the nerve injury, snap-frozen on dry ice, and stored at − 80 °C.

### Rat Transcriptomic and Bioinformatic Analysis

Total RNA from rat DH tissue was isolated using the Macherey–Nagel™ NucleoSpin™ RNA mini kit (Thermo Fisher, Waltham, MA USA) and labelled using an Ambion WT Expression kit (Life Technologies, Bleiswijk, The Netherlands) and hybridized to Affymetrix Rat Transcriptome Array (RTA) 1.0 (Affymetrix, Santa Clara, CA, USA). Sample labelling, hybridization to chips, and image scanning were performed according to the manufacturer’s instructions on an Affymetrix GeneTitan instrument by AROS Applied Biotechnology (Aarhus, Denmark). Quality control was performed using Affymetrix Expression Console and interpretation of data was facilitated by Affymetrix Transcriptome Analysis Console 2.0 (TAC2.0).

Transcripts exhibiting a fold change between the L5 SNL and sham surgery groups of ≥ 1.25 in absolute magnitude with corresponding *p*-values of < 0.05 obtained from a series of analyses of variance (ANOVA) were considered differentially expressed and suitable for further analysis and refinement. The transcriptomic data was further analysed using Ingenuity Pathway Analysis (IPA®) (Qiagen Hilden, Germany) [23]. IPA compares the transcriptomic expression profiles against the literature and allows the identification of any relationships to interacting systems or common molecular pathways. From the TAC2.0 and IPA analyses, nineteen genes of interest were tested in the human clinical samples by quantitative real-time polymerase chain reaction (qRT-PCR) and were selected according to their *p*-value and fold change in the rat transcriptomics, as well as their predicted molecular interactions based on the IPA analysis. These additional genes included *NLRP3*, *CASP3*, *CASP5, CASP8*, and *CASP9* based on their predicted molecular interactions with the human orthologues of the two significant rat caspase genes, *Casp1* and *Casp4* (Table 1).

**Table 1:** Gene expression changes in Sprague Dawley SNL vs. sham detected by Affymetrix transcriptomics array

Transcript cluster ID	Accession no	Gene symbol	Gene name	Fold change	*p-*value
TC0500001514.rn.1	NM_001080438.1	*A3galt2*	Alpha 1,3-galactosyltransferase 2	1.31	0.042
TC1500000736.rn.1	NM_012904	*Anxa1*	Annexin a1	1.43	0.047
TC0800000012.rn.1	NM_012762	*Casp1*	Caspase 1	1.27	0.046
TC0800000013.rn.1	NM_053736	*Casp4*	Caspase 4	1.34	0.025
TC0800001851.rn.1	NM_053960.3	*Ccr5*	Chemokine motif C–C Receptor 5	1.35	0.040
TC0400003743.rn.1	NM_012705	*Cd4*	CD4 molecule	1.26	0.049
TC1800001603.rn.1	NM_012839	*Cycs*	Cytochrome c; somatic like	1.33	0.033
TC0100004596.rn.1	NM_001005738.1	*Fpr2*	Formyl peptide receptor 2	− 1.35	0.002
TC1300000383.rn.1	NR_031899.1	*Mir-181b1*	MicroRNA 181-b1	− 1.4	0.012
TC0100006086.rn.1	NM_006189.1	*Omp*	Olfactory marker protein	− 1.25	0.005
TC1400000175.rn.1	NM_001130715	*Plac8*	Placenta-specific 8	1.61	0.02
TC0300002143.rn.1	NM_001195490.1	*Romo1*	Reactive oxygen species modulator 1	1.32	0.01
TC0500003800.rn.1	NM_031286	*Sh3bgrl3*	SH3 domain-binding glutamate-rich protein-like 3	1.28	0.008
TC1000003076.rn.1	NM_203411	*Tmem88*	Transmembrane protein 88	1.33	0.016
TC0500002825.rn.1	NM_053800	*Txn1*	Thioredoxin 1	1.30	0.003

*Mir-181b1* was not taken forward to the human analysis

### Human Clinical Samples

Fifty-one adult neuropathic pain patients were recruited from Seacroft Leeds Teaching Hospital. Questionnaires completed at the time of blood collection included screening questions for exclusion criteria for fibromyalgia and diabetes. Patient questionnaire data was collected, including the self-reported Leeds Assessment of Neuropathic Symptoms and Signs (S-LANSS), Patient Health Questionaire-9 (PHQ-9), and Graded Chronic Pain Scale (GCPS) which were completed by the patients. The PHQ-9 is used to monitor depression and response to anti-depressive treatment. The GCPS is indicative of pain intensity and disability severity. The higher the score, the more intense the pain and severe the pain-associated disability. The State-Trait Anxiety Inventory (STAI) was used to measure state anxiety and trait anxiety in scores STAI-Y1 and STAI-Y2 respectively. Clinical data including diagnosis, number of months since pain began, current medications, and comorbidities were also collected by questionnaire. For S-LANSS, the score is out of 24, where scores of ≥ 12 suggest pain of predominantly neuropathic origin. Nociceptive pain is defined by scores of < 12. PHQ-9 score is out of 27, with scores falling into one of five classifications with increasing depression severity. STAI-Y1 and STAI-Y2 scores are calculated from 20 questions, with scores ranging from 20 and 80, and higher scores indicating greater anxiety. Sixty-two control participants were recruited from the University of Huddersfield, UK. Neuropathic pain participants were age- and gender-matched against control participants.

The study was approved by the Yorkshire & The Humber – Bradford Leeds Research Ethics Committee (14/YH/0117) and adopted to the National Institute of Health Research Clinical Research Network (Portfolio ID: 16,774). Informed consent was obtained prior to participation and blood samples were collected using PAXgene Blood RNA Tubes (Qiagen) from all participants.

### RNA Extraction from Human Blood

Total RNA was extracted using the PAXgene RNA extraction kit according to the manufacturer’s instructions (Qiagen). In brief, the RNA was treated with DNase (Thermo Fisher Scientific) and purified on columns. The final RNA concentration was measured on a NanoDrop ND2000 ultraviolet–visible spectrophotometer (Labtech International Ltd, UK). Complementary DNA (cDNA) was prepared using 500 ng extracted RNA in a 10 µl reaction using the Verso cDNA Synthesis kit (Thermo Fisher Scientific) according to the manufacturer’s instructions.

### Quantitative Real-Time PCR

To analyse gene expression, qRT-PCR was performed on the Roche LC480 system (Roche Diagnostics Ltd, West Sussex, UK) in a 96-well format. A reaction mix of 10 µl per well was prepared with 1 µl diluted 1:50 cDNA, 0.3 µM forward and reverse primers, and 5 µl × 2 Roche Mastermix containing SYBR Green I dye (Roche Diagnostics Ltd). Cycling conditions were as follows: one cycle of preincubation at 95 °C for 5 min, 45 cycles of amplification including 10 s at 95 °C, 10 s at 60 °C, and 10 s at 72 °C, followed by the melting curve protocol of 5 s at 95 °C and 1 min at 65 °C, and finally cooling at 40 °C for 30 s. A GeNorm analysis was carried out on a random subset of participant samples to determine the most stable reference genes in the samples using qbase + software (Biogazelle, Gent, Belgium). Data was then normalised to the reference genes *TOP1* and *YWHAZ*, which met the stability criteria (average expression stability (M) value < 0.5, coefficient of variation (CV) < 25%). *TOP1* and *YWHAZ* primers were sourced from the Primerdesign human reference gene kit (Primerdesign Ltd, Southampton, UK).

### Statistical Analysis

Clinical data was summarized descriptively. The extent and pattern of missing data were noted, and any causes of data missingness was investigated.

#### Primary Analyses

Statistical analysis of clinical data was performed on normalised qRT-PCR gene expression data using comparing gene expression in neuropathic pain patients and controls, using a series of univariate main effects analyses of covariance (ANCOVA), controlling for age and gender. Uncorrected and Bonferroni-corrected significance (at the 5% significance level) was assessed, with the sensitivity of inferences of significance assessed by comparison of significance levels against Benjamini-Hochberg (B-H) critical values at FDR of 5%, 10%, and 25%. Significance according to Bonferroni-corrected *p*-values controls familywise error rate (the probability of making 1 or more false discoveries), and as such represents the method of most conservative inference.

#### Secondary Analyses

Four series of secondary analyses were conducted: an investigation of differential expression across groups in which the neuropathic pain group was subdivided into two contrasting pain groups; an investigation of differential expression across groups characterised by medications; receiver operating characteristic (ROC) analyses to assess the ability of gene combinations to effectively discriminative between the two pain groups; and correlational analyses on self-reported patient measures.

To determine how each differentially expressed gene may be contributing to a nociceptive or neuropathic pain component based on the S-LANSS score as opposed to the initial clinical assessment of neuropathic pain at recruitment, the cohort was dichotomized as *neuropathic pain* or *nociceptive pain*. Thirteen patients were assigned to the *nociceptive pain* group (< 12 S-LANSS score) and 38 to the neuropathic pain group (≥ 12 S-LANSS score). Further ANCOVA procedures were conducted on the expression levels, comparing controls against nociceptive pain patients against neuropathic pain patients. Additional ANCOVA procedures were conducted on gene expression amongst the neuropathic pain cohort according to the following medication groups: antidepressants, anticonvulsants, anti-inflammatories, and opioids. Significance was assessed in all secondary analyses using uncorrected *p*-values; corrections were informally applied if appropriate.

To investigate the hypothesis that gene expression levels may precede pain on the causal pathway, a series of multiple logistic regression analyses were conducted on neuropathic pain patients, considering the S-LANSS score, dichotomized as above. Twenty-three gene combinations were selected for analysis. Each combination was comprised of three genes from the set of those identified as being significantly differently expressed in controls and neuropathic pain patients. These gene combinations were selected manually according to their known functional similarities e.g. role in inflammation or apoptosis or based on significant levels in the clinical analyses. The combinations were limited to three genes per analysis as this should yield a sufficient level of predictive information without the pragmatic difficulties of selecting larger subsets, which would be likely to yield only a marginal improvement in predictive capability. For each combination, neuropathic pain patients (defined according to S-LANSS score) were compared against (i) nociceptive patients (excluding controls); (ii) controls (excluding nociceptive patients).

Predicted probabilities were saved from these analyses and used as test variables in a series of ROC analyses. For each analysis, the area under the ROC curve (AUROC) was calculated to assess discrimination capability. A 95% confidence interval was also calculated for the AUROC statistic, and the hypothesis that the true value of the AUROC statistic was 0.5 was tested at the 5% significance level. Optimum sensitivity and specificity were also derived, with an appropriate cut-off identified, using the closest-to-corner method, which defines the optimal cut-off point as that minimizing the Euclidean distance between the ROC curve and the idealized point corresponding to 100% sensitivity and specificity. The utility of each tested gene combination as a predictive tool was also assessed by calculation of the likelihood ratio at the point corresponding to optimum sensitivity and specificity.

All results from both sets of comparisons were tabulated. Additionally, ROC curves were generated for specific gene combinations of interest and yielded a maximum discriminatory capability (i.e. those yielding a score of ~ 0.9 or greater in the area under the ROC curve (AUROC)).

Relationships between patient-reported measures were assessed using correlational methods to obtain insight into trends that may occur amongst heterogeneous cases of neuropathic pain. The extent of baseline group imbalance on these measures was also assessed.

## Results

### Gene Expression Changes in Rat Transcriptome Array of Spinal Nerve Ligation Dorsal Horn Tissue

The expression of 1058 genes met the selection criteria (fold change ≥ 1.25 in absolute magnitude and *p*-value < 0.05); once the unannotated (unmapped) genes were removed, 193 genes remained that met these criteria. Of these 193, fifteen genes of interest were selected using both the criteria above and manually based on their known function relating to apoptosis, inflammation, and nervous system function (Table 1).

### Bioinformatic Analysis

**Fig. 1:**
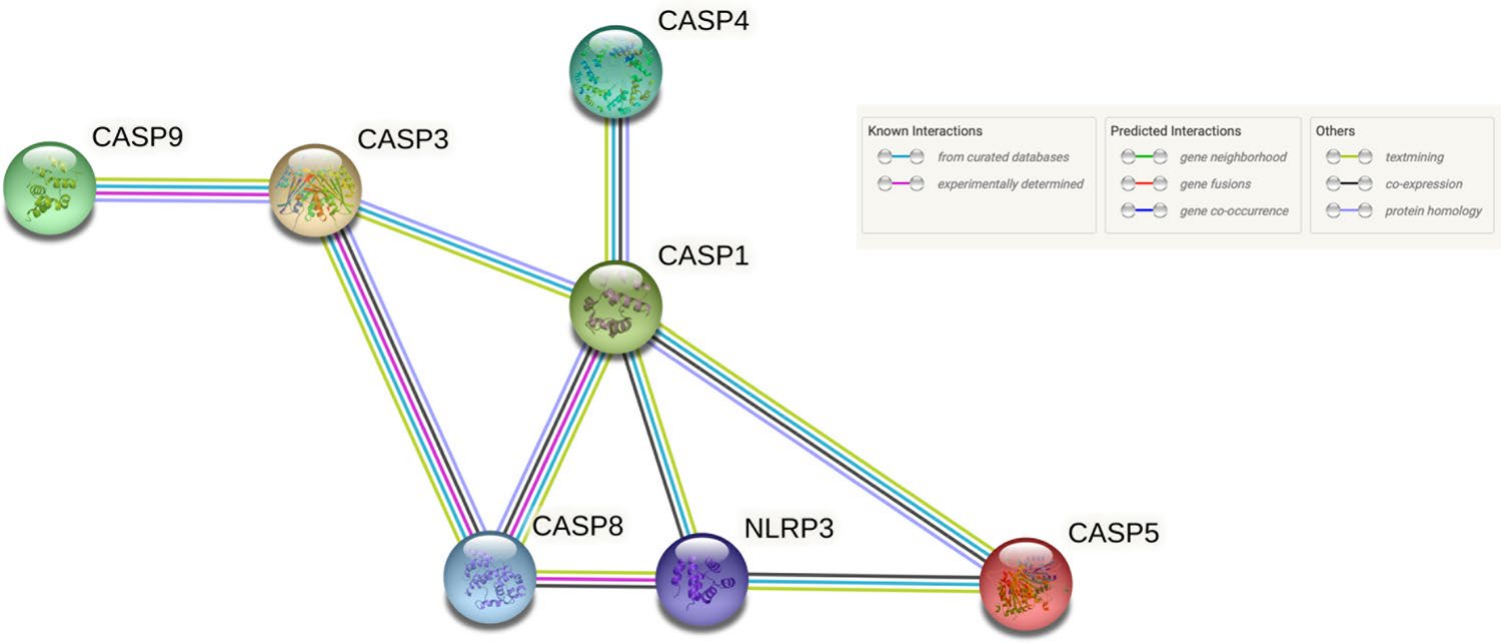
Human molecular network analysis of CASP1, CASP4, and CASP5 using String*.* A network analysis was performed with human CASP1, CASP4, and CASP5 using String (© String Consortium 2022). A minimum required interaction score of > 0.900 (very high confidence) was the criterion used to determine protein interaction, which is a culmination of evidence from known, predicted, and other interactions, and represented by different coloured edges

A bioinformatic analysis of the rat transcriptomic data using IPA® identified a network of genes, where their protein products are involved in inflammation and programmed cell death, including apoptosis (Cytochome c) [24] and pyroptosis (Caspase 1 and 4) [25] as well as the antioxidant factor Thioredoxin 1 [26], which also has an important role in the defence of neurons [27]. The genes *Cycs*, *Casp4*, *Casp1*, and *Txn1* were all upregulated in the SNL rat within the range of fold change 1.27–1.34; *p*-value < 0.05. These four molecules were found perturbed in the rat SNL. This represents a snapshot of the potentially complex and dynamic process of neuropathic pain. Pyroptosis is a highly inflammatory form of programmed cell death. Human Caspase 1 and mouse Caspase 11 (synonym Casp4) have been linked [28] and therefore considered inflammatory caspases. Other non-canonical roles are emerging for many caspases including apoptosis and synaptic plasticity [29, 30], and the dynamic of caspase molecules may be diverse. To briefly clarify, rat *Casp4*, based on the HGNC Comparison of Orthology Prediction (https://www.genenames.org), *CASP4 and CASP5* are the human orthologs, and *Casp11* (synonym *Casp4*) is the mouse ortholog. Due to the identification of two rat caspase genes (*Casp1* and *Casp4*) and the fact that *Casp4* pertains to the *CASP4* and *CASP5* human orthologues, a human network analysis of these three caspase molecules (CASP1, CASP4, and CASP5) was performed using String with a minimum required interaction score of > 0.900 (very high confidence), which is a culmination of evidence relating to their protein equivalents including co-expression and experimental/biochemical data as opposed to canonical function alone (Fig. 1). The analysis revealed a broader network of caspase proteins including CASP3, CASP8, CASP9, and NLRP3. Due to these interactions with the additional molecules, *CASP3*, *CASP8*, *CASP9*, and *NLRP3* were taken forward to the clinical gene expression analysis. *CASP5* has previously been tested in this clinical cohort [14], due to (A) the significant finding of the *CASP5* rat ortholog *Casp4* in the rat transcriptomic analysis and (B) the significant expansion of the clinical analysis including the AUROC analysis. We repeated the *CASP5* gene expression measure alongside the other candidates to encompass it within this extended analysis.

### Descriptive Summary of Clinical Cohort

The human orthologues of the genes identified in the rat transcriptome array (Table 1) and additional genes identified by the bioinformatic analysis were interrogated in a neuropathic pain clinical cohort. The cohort is summarized in Table 2 by patient type, and as a complete set. All values are given as a mean (SD). Gene expression is given as a measure of mRNA transcripts in patient blood samples by Calibrated Normalized Relative Quantity (CNRQ) calculated in qBase + software from qRT-PCR data from blood samples.

**Table 2:** Descriptive summary of cohort

Variable	Controls (*n* = 62)	Neuropathic pain patien*ts (n* = 51)	All (*n* = 113)
Age (years)	38.2 (13.6)	46.1 (12.6)	41.8 (13.7)
Pain duration (months)	Not applicable	95.7 (99.3)	95.7 (99.3) (*n* = 51)
S-LANSS score	Not applicable	15.4 (7.91)	15.4 (7.91) (*n* = 51)
PHQ-9 score	2.15 (2.34)	13.3 (7.28) (*n* = 50)	7.12 (7.58) (*n* = 112)
STAI-1 score	28.5 (8.24)	46.1 (13.4)	36.4 (13.9)
STAI-2 score	35.6 (9.19) (*n* = 51)	48.5 (13.1) (*n* = 23)	39.6 (12.1) (*n* = 74)
*A3GALT2* CNRQ	1.03 (0.854) (*n* = 38)	1.41 (1.01) (*n* = 38)	1.22 (0.948) (*n* = 76)
*ANXA1* CNRQ	1.01 (0.294) (*n* = 38)	1.11 (0.453) (*n* = 38)	1.06 (0.383) (*n* = 76)
*CASP1* CNRQ	0.980 (0.218) (*n* = 38)	1.07 (0.254) (*n* = 76)	1.03 (0.240) (*n* = 76)
*CASP3* CNRQ	1.03 (0.406) (*n* = 39)	1.06 (0.340) (*n* = 38)	1.05 (0.373) (*n* = 77)
*CASP4* CNRQ	0.958 (0.198) (*n* = 38)	1.08 (0.250) (*n* = 38)	1.02 (0.231) (*n* = 76)
*CASP5* CNRQ	0.886 (0.429) (*n* = 38)	1.42 (0.669) (*n* = 38)	1.15 (0.619) (*n* = 76)
*CASP8* CNRQ	0.939 (0.256) (*n* = 39)	1.18 (0.250) (*n* = 27)	1.04 (0.279) (*n* = 66)
*CASP9* CNRQ	0.844 (0.217) (*n* = 39)	1.26 (0.255) (*n* = 38)	1.05 (0.314) (*n* = 77)
*CCR5* CNRQ	0.984 (0.301) (*n* = 38)	1.19 (0.494) (*n* = 38)	1.08 (0.419) (*n* = 76)
*CD4* CNRQ	1.04 (0.348) (*n* = 38)	1.15 (0.271) (*n* = 38)	1.10 (0.315) (*n* = 76)
*CYCS* CNRQ	0.998 (0.155) (*n* = 38)	1.07 (0.304) (*n* = 38)	1.03 (0.242) (*n* = 76)
*FPR2* CNRQ	0.891 (0.292) (*n* = 38)	1.22 (0.396) (*n* = 38)	1.06 (0.383) (*n* = 76)
*NLRP3* CNRQ	1.04 (0.270) (*n* = 39)	1.03 (0.298) (*n* = 38)	1.03 (0.282) (*n* = 77)
*OMP* CNRQ	1.01 (0.640) (*n* = 31)	1.08 (1.28 (*n* = 40)	1.05 (1.05) (*n* = 71)
*PLAC8* CNRQ	1.57 (0.739) (*n* = 38)	2.03 (1.12) (*n* = 47)	1.92 (0.993) (*n* = 85)
*ROMO1* CNRQ	1.15 (0.247) (*n* = 38)	1.06 (0.412) (*n* = 38)	1.11 (0.340) (*n* = 76)
*SH3BGRL3* CNRQ	1.13 (0.261) (*n* = 38)	0.921 (0.209) (*n* = 38)	1.02 (0.256) (*n* = 76)
*TMEM88* CNRQ	0.881 (0.432) (*n* = 38)	1.38 (0.519) (*n* = 38)	1.13 (0.535) (*n* = 76)
*TXN1* CNRQ	1.19 (0.742) (*n* = 34)	1.28 (0.809) (*n* = 41)	1.23 (0.774) (*n* = 75)

*CNRQ*, Calibrated Normalized Relative Quantity calculated in qBase + software; S-LANSS score is out of 24; PHQ-9 score is out of 27; STAI-1 and STAI-2 scores are out of 80. All values given as mean (SD)

### Primary Analysis: Differential Gene Expression Changes Between Clinical Neuropathic Pain Patients and Controls

Gene expression analysis in human samples included fourteen of the fifteen significant rat genes (*Mir-181b1* was excluded and not pursued further in this analysis). Uncorrected, Bonferroni-corrected *p*-values, and FDR-adjusted critical values using the Benjamini-Hochberg (B-H) method (assuming FDRs of 5%, 10%, and 25%) associated with the ANCOVA models conducted on all genes analysed in the rat model, plus those identified as being of interest based on their interactions with these genes, are summarized in Table 3 below. All models were corrected for age and gender.

**Table 3:** Gene expression changes in whole blood of neuropathic pain patients (whole cohort) versus control patients

Gene	*p*-value from SNL rat model	Clinical fold change	Uncorrected *p*-value	Rank (*m* = 19)	Bonferroni-corrected *p*-value	B-H critical value^1^	B-H critical value^2^	B-H critical value^3^
*A3GALT2*	0.0421	1.37	0.051	9	0.969	0.0237	0.0474	0.118
*ANXA1*	0.0477	1.10	0.273	14	1.00	0.0368	0.0736	0.184
*CASP1*	0.0459	1.10	0.090	11	1.00	0.0289	0.0578	0.145
*CASP3*	-	1.03	0.648	18	1.00	0.0474	0.0948	0.237
*CASP4*	0.0252	1.10	**0.017**	7	0.323	0.0184	0.0368	0.0921
CASP5*	**-**	**1.57**	**0.000119**	**3**	**0.00226**	**0.00789**	**0.0158**	**0.0394**
CASP8*	**-**	**1.79**	**0.000309**	**5**	**0.00587**	**0.0132**	**0.0264**	**0.0658**
CASP9*	**-**	**1.67**	**1.101 × 10**^**−10**^	**1**	**2.09 × 10**^**−9**^	**0.00263**	**0.00526**	**0.0131**
*CCR5*	0.0403	1.23	**0.030**	8	0.570	0.0211	0.0422	0.105
*CD4*	0.0499	1.11	0.125	12	1.00	0.0316	0.0632	0.158
*CYCS*	0.0334	1.08	0.220	13	1.00	0.0342	0.0684	0.171
*FPR2*	**0.0024**	**1.33**	**0.000145**	**4**	**0.00278**	**0.0105**	**0.0210**	**0.0526**
*NLRP3*	-	1.01	0.831	19	1.00	0.0500	0.100	0.250
*OMP*	0.0048	1.03	0.555	17	1.00	0.0447	0.0894	0.224
*PLAC8*	0.0246	1.08	0.080	10	1.00	0.0263	0.0526	0.132
*ROMO1*	0.0136	− 1.08	0.312	15	1.00	0.0395	0.0790	0.197
SH3BGRL3*	**0.0085**	− **1.19**	**0.000333**	**6**	**0.00633**	**0.0158**	**0.0316**	**0.0789**
TMEM88*	**0.0167**	**1.56**	**0.000020**	**2**	**0.00038**	**0.00526**	**0.0105**	**0.0263**
*TXN1*	0.0034	1.03	0.425	16	1.00	0.0421	0.0824	0.211

^1^FDR = 0.05

^2^FDR = 0.10

^3^FDR = 0.25

^*^Genes significant in all statistical rubrics are in bold

Hence using uncorrected *p*-values, eight genes (*CASP4, CASP5, CASP8, CASP9, CCR5, FPR2, SH3BGRL3,* and *TMEM88*) were found to be significantly differentially expressed in the clinical cohort, six of these genes reverse-translated from the rat SNL model representing cross-species validation to clinical cases of neuropathic pain, with strong evidence for the role of these genes in the pathophysiology of neuropathic pain. Six of the eight genes (*CASP5, CASP8, CASP9, FPR2, SH3BGRL3,* and *TMEM88*) remained significant considering Bonferroni-corrected *p*-values.

Under an FDR of 5%, seven genes (*CASP4, CASP5, CASP8, CASP9, FPR2, SH3BGRL3,* and *TMEM88*) were found to be significantly differentially expressed in the clinical cohort. It would be expected that about 5% of these genes (i.e. about 0 or 1) would be false discoveries. Allowing an FDR of 10% additionally resulted in gene *CCR5* being declared positive (total of eight genes). It would be expected that about 10% of these genes (i.e. ~ 1) would be false discoveries. Allowing an FDR of 25% additionally resulted in gene *A3GALT2, CASP1, CD4,* and *PLAC8* (i.e. twelve genes in total) being declared positive. It would be expected that about 25% of these genes (i.e. ~ 3) would be false discoveries. However, whilst FDR analysis is essential to ensure for statistical robustness, it must be noted that these genes were not randomly chosen but came from a rat model of neuropathic pain and associated bioinformatic analysis that were subsequently assessed in human neuropathic pain patients.

**Fig. 2:**
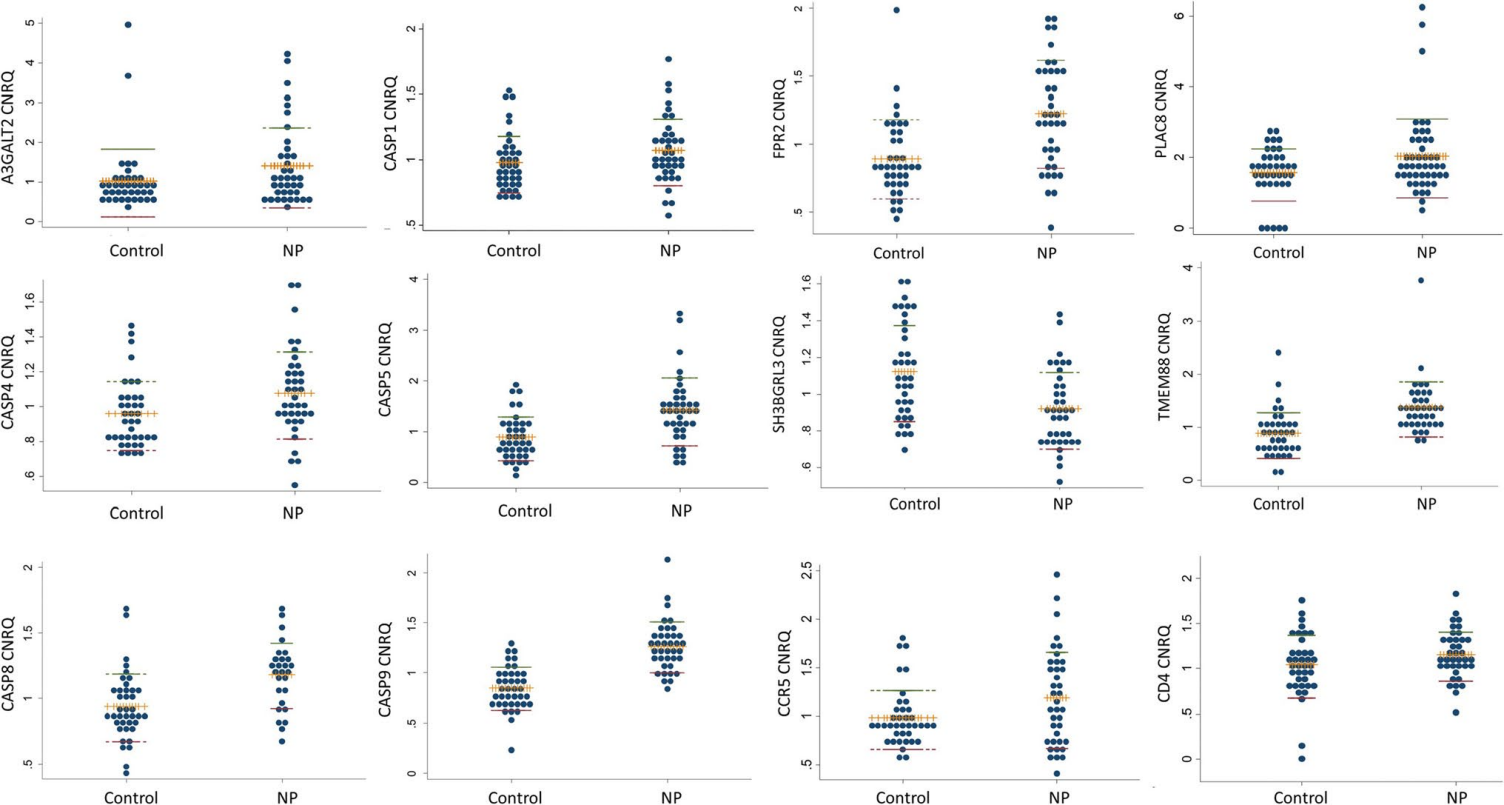
Expression changes in blood samples from neuropathic pain patients versus non-neuropathic pain controls with FDR correction and controlling for age and gender. Normalised relative quantity (NRQ) of differential expression in controls and neuropathic pain (NP) patients for all genes declared significant under any rubric. Horizontal lines through grouped data denote mean values and standard deviations

Of the genes found to be significantly differentially expressed in the clinical cohort, *A3GALT2, CASP1, CASP4, CASP5, CASP8, CASP9, CCR5, CD4, FPR2, PLAC8,* and *TMEM88* were significantly greater in neuropathic pain patients than controls, whereas expression of *SH3BGRL3* was significantly less in neuropathic pain patients versus controls. Genes that were declared significant in all rubrics from Table 3 are illustrated in Fig. 2.

### Secondary Analysis (1): Gene Expression in Controls and Patients with Neuropathic and Nociceptive Pain

In the comparison of gene expression across patient groups in which neuropathic pain patients were reclassified based on the S-LANSS questionnaire as nociceptive pain (S-LANSS score < 12) or neuropathic pain (S-LANSS score ≥ 12), nine genes (*CASP4, CASP5, CASP8, CASP9, CCR5, FPR2, PLAC8, SH3BGRL3,* and *TMEM88*) were found to be significantly differentially expressed across the three clinical cohort groups. This set of genes is comprised of the same set of eight genes which were significantly differentially expressed over the 2-group analysis, plus the single additional gene *PLAC8*. Mean values and standard deviations, plus uncorrected *p*-values arising from comparisons of both pain groups against the control group, are summarized in Table 4. All analyses are controlled for age and gender.

**Table 4:** Gene expression levels (mean (SD)) and significance levels of genes declared significantly differentially expressed across patient groups differentiated by pain level (S-LANSS score)

Gene	Patient group				Uncorrected *p*-values^1^	
	Controls	Nociceptive pain	Neuropathic pain	All	Nociceptive pain	Neuropathic pain
*CASP4*	0.958 (0.198) (*n* = 38)	0.926 (0.228) (*n* = 9)	1.12 (0.241) (*n* = 29)	1.02 (0.231) (*n* = 76)	0.899	**0.00301**
*CASP5*	0.886 (0.429) (*n* = 38)	1.18 (0.483 (*n* = 9)	1.49 (0.707) (*n* = 29)	1.15 (0.619) (*n* = 76)	0.192	**0.0000450**
*CASP8*	0.939 (0.256) (*n* = 39)	1.28 (0.252) (*n* = 8)	1.14 (0.242) (*n* = 19)	1.04 (0.279) (*n* = 66)	**0.000595**	**0.00572**
*CASP9*	0.844 (0.217) (*n* = 39)	1.29 (0.176) (*n* = 9)	1.25 (0.276) (*n* = 29)	1.05 (0.314) (*n* = 77)	**1.00 × 10** ^**-5**^	**1.74 × 10** ^**-9**^
*CCR5*	0.984 (0.301) (*n* = 38)	0.901 (0.411) (*n* = 9)	1.28 (0.489) (*n* = 29)	1.08 (0.419) (*n* = 76)	0.557	**0.00355**
*FPR2*	0.891 (0.292) (*n* = 38)	1.26 (0.379) (*n* = 9)	1.21 (0.407) (*n* = 29)	1.06 (0.383) (*n* = 76)	**0.012**	**0.000490**
*PLAC8*	1.57 (0.739) (*n* = 38)	2.75 (1.69) (*n* = 14)	1.73 (0.569) (*n* = 33)	1.92 (0.993) (*n* = 85)	**0.000325**	0.473
*SH3BGRL3*	1.13 (0.261) (*n* = 38)	0.977 (0.206) (*n* = 9)	0.904 (0.210) (*n* = 29)	1.02 (0.256) (*n* = 76)	0.0774	**0.000397**
*TMEM88*	0.881 (0.432) (*n* = 38)	1.46 (0.401) (*n* = 9)	1.35 (0.554) (*n* = 29)	1.13 (0.535) (*n* = 76)	**0.00106**	**0.000175**

^1^Reference category Control; significant values (*p* < 0.05) are indicated in bold

### Secondary Analysis (2): Gene Expression in Patients Differentiated by Medication Groups

Gene expression amongst the neuropathic pain cohort was analysed according to the following medication groups: antidepressants, anticonvulsants, anti-inflammatories, and opioids. *A3GALT2*, *PLAC8*, and *ROMO1* were differentially expressed in patients taking opioid medication versus patients not taking opioid medication. Compared with patients not taking opioid medication, expression of *A3GALT2 and PLAC8* was reduced, and expression of *ROMO1* was increased in patients taking opioid medication. *FPR2* was differentially expressed in patients taking anti-inflammatory medication, with expression reduced in patients taking anti-inflammatory medication. No other genes showed differential expression with respect to any of the medication groups. Parameters of significantly differentially expressed genes are summarized in Table 5.

**Table 5:** Gene expression levels (mean (SD)) and significance levels of genes declared significantly differentially expressed across patient groups differentiated by medication groups

Gene	Non-opioid	Opioid	All	Uncorrected *p*-value
*A3GALT2*	2.02 (1.09) (*n* = 19)	0.815 (0.348) (*n* = 18)	1.43 (1.01) (*n* = 37)	4.00 × 10^−6^
*PLAC8*	2.62 (1.39) (*n* = 17)	1.69 (0.783) (*n* = 30)	2.03 (1.12) (*n* = 47)	0.00720
*ROMO1*	0.881 (0.326) (*n* = 19)	1.20 (0.385) (*n* = 18)	1.04 (0.387 (*n* = 37)	0.0116
Gene	Non anti-inflammatory	Anti-inflammatory	All	Uncorrected ***p*-**value
*FPR2*	1.35 (0.365) (*n* = 22)	1.09 (0.351) (*n* = 15)	1.24 (0.378) (*n* = 37)	0.0356

### Secondary Analyses (3): ROC Analyses of Gene Combinations as Pain Predictors

From the differential expression analysis, the combinations of genes that were tested in ROC analyses for their capability to be discriminative between controls and patients with neuropathic pain are summarized in Supplementary Table 2. The number of genes in these analyses was limited to three genes per analysis as outlined in the methods.

**Fig. 3:**
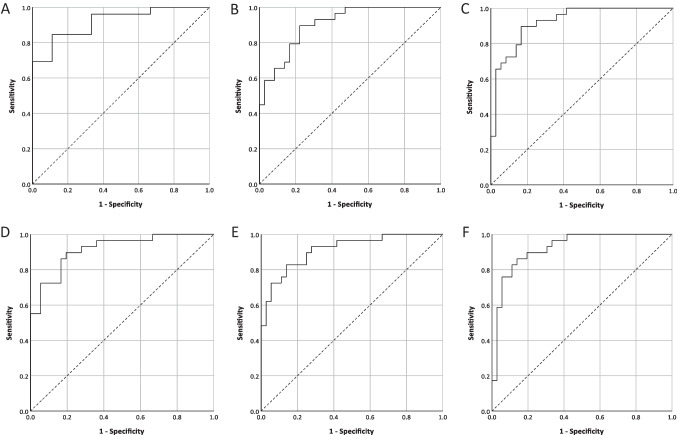
ROC curves comparing groups. ROC curves for **A** neuropathic versus nociceptive pain (< 12 S-LANSS score), disregarding controls for gene combination 17 – *PLAC8*, *ROMO1*, and *A3GALT2*. **B**–**F** neuropathic pain versus controls, disregarding nociceptive pain (< 12 S-LANSS score) for respective gene combinations 7 – *CASP4*, *CASP5*, *CASP9*; 10 – *CASP5*, *CASP9*, *TMEM88*; 12 – *CASP5*, *CASP9*, *FRP2*; 15 – *CASP9*, *FPR2*, *TMEM88*; 19 – *CASP4*, *CCR5*, *SH3BGRL3*; and 23 – *CASP9*, *SH3BGRL3*, *TMEM88*

The areas under each ROC curve (AUROC), with 95% confidence intervals and the significance level of the null hypothesis that AUROC = 0.5, appear in Table 6 (comparing patients with neuropathic pain and patients with nociceptive pain (S-LANSS < 12)) and Table 7 (comparing patients with neuropathic pain and controls), alongside the value of the predictive probability associated with an optimum combination of sensitivity and specificity, with values of sensitivity, specificity, and likelihood ratio (LR) given at that point. Combinations (those with good discriminatory powers as measured by the AUROC statistic) are highlighted. Figure 3 illustrates the ROC curves for each of the highlighted gene combinations identified in Tables 6 and 7.

**Table 6:** ROC analyses parameters for neuropathic versus nociceptive pain (< 12 S-LANSS score) disregarding controls

Combination	AUROC	95% CI for AUROC	*p*-value^1^	*P*_OPT_^2^	Sensitivity at *P*_OPT_	Specificity at *P*_OPT_	LR at *P*_OPT_
1	0.686	(0.512, 0.859)	0.096	0.496	0.517	0.889	4.66
2	0.713	(0.516, 0.909)	0.057	0.695	0.793	0.556	1.79
3	0.625	(0.427, 0.822)	0.264	0.721	0.828	0.444	1.49
4	0.762	(0.598, 0.927)	0.019	0.775	0.724	0.889	6.52
5	0.782	(0.573, 0.991)	0.013	0.679	0.923	0.667	2.77
6	0.838	(0.671, 1.00)	0.003	0.730	0.846	0.778	3.81
7	0.720	(0.525, 0.915)	0.048	0.662	0.828	0.556	1.86
8	0.783	(0.575, 0.991)	0.022	0.667	0.789	0.750	3.16
9	0.704	(0.484, 0.924)	0.100	0.684	0.674	0.750	2.74
10	0.670	(0.485, 0.856)	0.095	0.760	0.655	0.667	1.97
11	0.704	(0.474, 0.934)	0.100	0.688	0.632	0.750	2.53
12	0.659	(0.442, 0.876)	0.154	0.756	0.759	0.667	2.28
13	0.724	(0.492, 0.955)	0.071	0.686	0.684	0.750	2.74
14	0.697	(0.463, 0.932)	0.111	0.677	0.789	0.750	3.16
15	0.667	(0.468, 0.866)	0.135	0.763	0.655	0.778	2.95
16	0.663	(0.444, 0.881)	0.144	0.734	0.793	0.667	2.38
17*	**0.919**	**(0.827, 1.000)**	** < 0.001**	**0.664**	**0.846**	**0.889**	**7.62**
18	0.778	(0.597, 0.959)	0.013	0.798	0.690	0.889	6.21
19	0.816	(0.642, 0.991)	0.005	0.702	0.828	0.778	3.73
20	0.805	(0.611, 0.998)	0.006	0.748	0.793	0.778	3.57
21	0.724	(0.527, 0.922)	0.045	0.676	0.828	0.556	1.86
22	0.644	(0.450, 0.838)	0.198	0.808	0.483	0.778	2.17
23	0.632	(0.441, 0.823)	0.236	0.691	0.828	0.44	1.49

^1^Testing the hypothesis that AUROC = 0.5

^2^Predictive probability associated with optimum combinations of sensitivity and specificity (using the closest-to-corner method)

^*^Combinations (those with good discriminatory powers as measured by the AUROC statistic) are in bold

**Table 7:** ROC analyses parameters for neuropathic versus controls disregarding pain cases with < 12 S-LANSS score

Combination	AUROC	95% CI for AUROC	*p*-value^1^	*P*_OPT_^2^	Sensitivity at *P*_OPT_	Specificity at *P*_OPT_	LR at *P*_OPT_
1	0.852	(0.794, 0.950)	< 0.001	0.572	0.759	0.921	9.61
2	0.772	(0.655, 0.890)	< 0.001	0.464	0.690	0.816	3.74
3	0.829	(0.728, 0.930)	< 0.001	0.388	0.828	0.769	3.49
4	0.792	(0.694, 0.910)	< 0.001	0.472	0.655	0.892	6.22
5	0.881	(0.774, 0.988)	< 0.001	0.527	0.808	0.857	5.65
6	0.815	(0.686, 0.944)	< 0.001	0.506	0.808	0.762	3.39
7*	**0.904**	**(0.835, 0.973)**	** < 0.001**	**0.378**	**0.897**	**0.778**	**4.03**
8	0.839	(0.736, 0.942)	< 0.001	0.393	0.684	0.833	4.11
9	0.923	(0.855, 0.990)	< 0.001	0.430	0.842	0.833	5.05
10*	**0.922**	**(0.860, 0.985)**	** < 0.001**	**0.465**	**0.897**	**0.833**	**5.38**
11	0.873	(0.783, 0.962)	< 0.001	0.223	0.895	0.694	2.93
12*	**0.916**	**(0.849, 0.983)**	** < 0.001**	**0.470**	**0.833**	**0.862**	**5.17**
13	0.839	(0.733, 0.946)	< 0.001	0.231	0.842	0.694	2.76
14	0.835	(0.721, 0.949)	< 0.001	0.303	0.842	0.778	3.79
15*	**0.913**	**(0.844, 0.981)**	** < 0.001**	**0.440**	**0.828**	**0.861**	**5.96**
16	0.839	(0.746, 0.932)	< 0.001	0.437	0.759	0.816	4.12
17	0.645	(0.481, 0.808)	0.091	0.503	0.808	0.476	1.54
18	0.886	(0.801, 0.971)	< 0.001	0.510	0.793	0.895	7.53
19	0.860	(0.769, 0.951)	< 0.001	0.568	0.690	0.921	8.74
20	0.801	(0.694, 0.908)	< 0.001	0.481	0.621	0.895	5.90
21	0.865	(0.772, 0.957)	< 0.001	0.451	0.828	0.816	4.50
22	0.869	(0.781, 0.958)	< 0.001	0.411	0.862	0.789	4.10
23*	**0.923**	**(0.859, 0.988)**	** < 0.001**	**0.451**	**0.862**	**0.861**	**6.21**

^1^Testing the hypothesis that AUROC = 0.5

^2^Predictive probability associated with optimum combinations of sensitivity and specificity (using the closest-to-corner method)

^*^Combinations (those with good discriminatory powers as measured by the AUROC statistic) are in bold

Table 6 reveals that ten gene combinations yielded AUROC scores representing discriminatory ability significantly better than chance. The single-gene combination number 17 (*PLAC8*, *ROMO1*, *A3GALT2*) achieved the highest level of discriminatory power (AUROC = 0.919) and yielded the highest likelihood ratio (LR) of any of the combinations tested on this comparison of groups (LR = 7.62) (Fig. 3A). This value of the LR is obtained using a cut-off point (*P*_*OPT*_) of 0.664, indicating that classifying cases for whom a predicted probability of greater than 0.664 from a multiple logistic regression analysis of pain type on *PLAC8*, *ROMO1*, and *A3GALT2* expression levels is obtained, yielding optimum discrimination. The high LR value implies a test of high utility, with a predicted probability of above 0.664 for a particular patient being 7.62 times more likely in a patient with neuropathic pain than a patient with nociceptive pain. This could provide a valuable test for discriminating neuropathic versus mixed pain amongst patients diagnosed as having either neuropathic or nociceptive pain. Other combinations of genes achieving high levels of discriminatory capability with respect to this patient group comparison include combination number 6 (*SH3BGRL3*, *TMEM88*, *PLAC8*) which achieved an AUROC of 0.838; and combination number 19 (*CASP4*, *CCR5*, *SH3BGRL3*) which achieved an AUROC of 0.816. Combination number 4 (*FPR2*, *CCR5*, *CD4*) yielded a high level of utility (LR = 9.52) but moderate discriminatory ability. No single gene is featured in all the high-value combinations with respect to this patient group comparison; however, *PLAC8* and *SH3BGRL3* both appear in two of the three combinations with the highest discriminatory capability.

Table 7 reveals that 22 gene combinations yield AUROC scores representing discriminatory ability significantly better than chance. Several gene combinations yield high levels of discriminatory power, with 5 combinations achieving AUROC statistics in excess of 0.900. Combination number 23 (*SH3BGRL3*, *TMEM88*, *CASP9*) achieved the highest level of discriminatory power (AUROC = 0.923) (Fig. 3F). Other combinations yielding AUROC statistics in excess of 0.900 included combination numbers 7 (*CASP4*, *CASP5*, *CASP9*); 10 (*CASP9*, *CASP5*, *TMEM88*); 12 (*CASP9*, *CASP5*, *FPR2*); and 15 (*TMEM88*, *FPR2*, *CASP9*) (Fig. 3B–E). The *CASP9* gene is common to all these combinations, and the *CASP5* and *TMEM88* genes both appear in three out of the five listed combinations. All the above combinations may be useful as tests of neuropathic pain (comparing against control patients). Combination number 23 also yielded the highest likelihood ratio statistics of the above subset of combinations (LR = 6.21, obtained at a cut-off value of 0.451). Hence, classifying cases for whom a predicted probability of greater than 0.451 from a multiple logistic regression analysis of neuropathic pain on *SH3BGRL3*, *TMEM88*, and *CASP9* expression levels as neuropathic yields optimum discrimination. The LR value implies that a predicted probability of greater than 0.451 obtained from a particular patient is 6.21 times more likely in a patient with neuropathic pain than in a control patient.

All other gene combinations listed above also yielded high LR values. However, high LR values were also yielded from combinations with lower discriminatory power. For example, combination 1 (*A3GALT2*, *SH3BGRL3*, *TMEM88*) and combination 19 (*CASP4*, *CCR5*, *SH3BGRL3*), which yielded likelihood ratios of 9.61 and 8.74, respectively, the highest LR values obtained by any combination with respect to this comparison. The interpretation of the high LR values is as above.

### Secondary Analyses (4): Correlational Analysis of Patient-Reported Measures

PHQ-9 depression scores were strongly positively correlated with GCPS (*r* = 0.715, *p* < 0.001) amongst neuropathic pain patients. Amongst neuropathic pain and control patients, PHQ-9 scores were also strongly correlated with STAI-Y1 scores for state anxiety (*r* = 0.757, *p* < 0.001), and STAI-Y2 for trait anxiety (*r* = 0.786, *p* < 0.001). STAI-Y1 and STAI-Y2 were also strongly significantly positively correlated (*r* = 0.803, *p* < 0.001).

Both PHQ-9 and the STAI1 and STAI2 scores differed substantially across patient groups, with the PHQ-9 score in particular being lower by a factor of in controls. Mean PHQ-9 scores were 2.15 (SD 2.34) in controls and 13.3 (SD 7.28) in neuropathic pain patients (Table 2). Smaller substantive differences were observed on the STAI instruments, with mean STAI1 and STAI2 scores of 28.5 (SD 8.24) and 35.6 (SD 9.19) in controls and 46.1 (SD 13.4) and 48.5 (SD 13.1) in neuropathic pain patients.

All self-reported patient questionnaire instruments (PHQ-9, GCPS, STAI1, and STAI2) were significantly mutually correlated, with measured correlations ranging from moderately strong positive (*r* = 0.410) for the relationship between GCPS and STAI1, to very strong positive (*r* = 0.803) for the relationship between STAI1 and STAI2, which is expected and provides confidence in the collection of the data. All correlations were statistically significant. The positive correlation between measures such as the GCPS and PHQ-9 score is not surprising as the association between chronic pain and depression is well established [31]. PHQ-9, GCPS, STAI1, and STAI2 will not be discussed any further.

## Discussion

In this study, we have applied a reverse translational approach, including cross-tissue analysis, that has provided us with insights into molecular pathways altered in neuropathic pain (rat model), but also how neuropathic pain may manifest in the human condition. We have used human blood in this study with (A) the purpose of identifying perturbed molecular changes as a reflection of what might be occurring in the central nervous system, as blood–brain barrier permeability is modulated in chronic pain [20, 21]; and (B) a tissue that can be readily accessed and tested in the clinic, where molecular perturbations can be easily detected to help delineate the variability in neuropathic pain diagnosis and form the basis of a more robust biomarker test.

Fifteen genes were identified as significantly differentially expressed in the dorsal horn of the rat L5 SNL neuropathic pain model (Table 1). Of these fifteen, fourteen were taken forward to the primary clinical analysis. Four additional genes were identified through a bioinformatical analysis and one gene, *CASP5*, was included as *CASP4*/*CASP5* are the human orthologs of rat *Casp4* (*mouse Casp11*), equating to a total of nineteen genes in the clinical analysis. Of the nineteen genes analysed in the clinical cohort, eight were significantly differentially expressed using uncorrected *p*-values (*CASP4, CASP5, CASP8, CASP9, CCR5, FPR2, SH3BGRL3,* and *TMEM88*). Excluding *CASP8* and *CASP9*, six of these genes represented a cross-species validation from the rat SNL model to clinical cases of neuropathic pain suggesting strong evidence for the role of these genes in the pathophysiology of neuropathic pain. Six genes (*CASP5, CASP8, CASP9, FPR2, SH3BGRL3,* and *TMEM88*) remained significant after a series of rigorous *p*-value corrections (Bonferroni/FDR) (Table 3). These genes represent potential biomarkers of neuropathic pain and warrant further investigation.

The caspase pathways involve cascades of zymogen activation which trigger either inflammation or apoptosis, depending on the caspases involved. Inflammatory caspase activity (Caspases 1 and 4/5) in the neuropathic pain phenotype may increase inflammatory mediators in neuropathic pain (see review [32]). Regarding *CASP1* and *CASP4*, although significantly altered in the rat SNL model (Table 1), only *CASP4* was significant in the clinical analysis (*p* = 0.017), but not after Bonferroni/FDR corrections were applied (Table 3). *CASP5*, along with *CASP4*, is the human ortholog to rat *Casp4* (mouse *Casp11*) and remained significant across all rubrics and was upregulated 2.33-fold in neuropathic pain patients, which validates a previous finding in an independent cohort [15]. The caspase pathways likely to be active in the clinical cohort are indicated by the upregulation of apoptosis initiators *CASP8* and *CASP9* (Table 3, fold change = 1.79 and 1.67 respectively) and significant across all statistical rubrics (Table 3, Bonferroni-corrected *p* = 0.00587 and *p* = 2.09 × 10^−9^ respectively). Caspase 8 is activated via the extrinsic pathway [33], which via cytochrome c release and caspase 9 causes cell death [34]. Upregulation of these genes suggests that apoptosis pathways are active in neuropathic pain, and detectable in blood samples. *CASP9* was the most significant gene differentially expressed in the clinical analysis and was also most apparent in the AUROC analysis (see below). Caspase 9 has been demonstrated to have multiple functions, including cellular differentiation and proliferation [35], maturation of sensory neurons [36], and mitochondrial homeostasis [37]. Caspase 9 has been linked to chronic neurodegeneration and neuronal injury, with significantly increased Caspase 9 expression, including vertebral disk degeneration severity, and thus could represent a useful therapeutic target [38, 39]. Moreover, a Caspase 9 promoter polymorphism that substantially enhances the transcriptional activity of the *CASP9* gene has been significantly associated with discogenic lower back pain [40], a finding which has been replicated [41, 42].

Three other genes significant across all the statistical rubrics included *FPR2*, *SH3BGRL3*, and *TMEM88* (Table 3). *FPR2* codes for the G-protein coupled receptor formyl peptide receptor 2 (FPR2) involved in the attenuation of the inflammation response [43] and limits tissue damage by neutrophil response attenuation [44, 45]. *Fpr2* is downregulated in the rat model (fold change =  − 1.35, *p* = 0.002) and increased in neuropathic pain patients (fold change = 1.33, Bonferroni-corrected *p* = 0.00278). FPR2 has several known ligands including Annexin A1 and lipoxin A4. Annexin A1 is a Ca^2+^-dependent phospholipid-binding protein which suppresses eicosanoid production and inhibits leukocyte adhesion in the acute phase of inflammation (Perretti & Dalli, 2009). Lipoxin A4 is a product of arachidonic acid and suppresses the expression of pro-inflammatory genes (Chandrasekharan & Sharma-Walia, 2015). Increased expression of *FPR2* is indicative of active anti-inflammatory pathways, supported by the concurrent upregulation of its ligand, *Anxa1*, with a fold change of 1.43 (*p* = 0.0470) in rats (Table 1). This interaction modulates neutrophil recruitment and activates neutrophil apoptosis [46]. The downregulation of its inhibitory microRNA, miR-181b1 (fold change =  − 1.4, *p*-value = 0.012), suggests this *Fpr2* expression inhibitory mechanism is not active in the SNL model.

The *SH3BGRL3* gene codes for the SH3 domain–binding glutamic acid–rich-like protein 3 were upregulated in the rat SNL model (fold change = 1.30, *p* = 0.0085) and downregulated (fold change =  − 1.19, Bonferroni-corrected *p* = 0.00663) in the clinical cohort. SH3BGRL3 is also known as tumour necrosis factor-alpha inhibitory protein, TIP-B1 [47], and is thioredoxin-like but cannot reduce other proteins, as it lacks the CXXC motif [48]. Differential expression of this gene supports other evidence for active anti-inflammatory mechanisms in neuropathic pain, which has been reported by previous studies [49, 50].

*TMEM88* codes for target transmembrane protein 88, which was upregulated in the SNL model (fold change = 1.33, *p* = 0.016) and in neuropathic pain patients (fold change = 1.56, Bonferroni-corrected *p* = 0.00038). TMEM88 is a known inhibitor of the Wnt/β-catenin canonical pathway, which is involved in neural development and plasticity in embryogenesis and in adult brains [51], and has been implicated in the production of hyperalgesia and allodynia in rat models of neuropathic pain [52, 53]. *PLAC8* codes for the placenta-specific 8 protein and can significantly activate the Wnt/β-catenin signalling pathway [54]. *Plac8* was found upregulated in the SNL model (fold change = 1.61, *p* = 0.0246), but only trending in the primary analysis (*p* = 0.080). However, a secondary analysis grouping pain patients by S-LANSS scores showed *PLAC8* to be significantly upregulated in the nociceptive group (*p* = 00,035) and not the neuropathic group (*p* = 0.473) according to the S-LANSS scores (Table 4). In contrast, *TMEM88* was significant in both S-LANSS groups. Given the role of Wnt/β-catenin signalling in neuronal plasticity and pain, both *TMEM88* and *PLAC8* may represent opposing regulatory pathways active in neuronal remodelling, which is known to be an important element in neuropathic pain development [55].

Secondary outcomes included investigating gene expression in patients differentiated by medication groups (Table 5). The most notable finding in this analysis was that *A3GALT2*, which was found upregulated in the rat SNL model (fold change = 1.31, *p* = 0.0421) but not significant in the primary clinical analysis, was significantly associated with patients on opioid medication (uncorrected *p* = 4.00 × 10^−6^). From Table 5, patients that were not taking opioids had a twofold increase in *A3GALT2* compared to controls, correlating with what was identified in the rat neuropathic pain model (Table 1), where those taking opioids had a slight reduced expression compared to controls. Therefore, it would appear that *A3GALT2* is dysregulated in neuropathic pain, and that *A3GALT2* levels are normalised with opioid treatment, which could relate to not only opioid function in neuropathic pain, but also a potential avenue for therapeutic development. In the context of neuropathic pain, *A3GALT2* upregulation might be indicative of increased activation of natural killer [NK] cells, as its isoglobotrihexosylceramide product (iGb3) is recognised by NK cells [56]. NK cells have been reported to play a potential protective role in neuropathic pain and may service as a marker for pain chronicity [57].

As discussed, a secondary analysis, grouping the patients by S-LANSS (neuropathic vs nociceptive), was performed on all patients. Genes significantly differentially expressed (considering uncorrected *p*-values) exclusively in the high S-LANSS score group (indicative of neuropathic pain) included *CASP4, CASP5, CCR5, and SH3BGRL3* (Table 4). *CASP9* was significant in both groups, but there was a clear, discernible, significant difference in the high S-LANSS compared to the low S-LANSS (*p* = 1.74 × 10^−9^ vs *p* = 1.00 × 10^−5^ respectively). As discussed above, this could relate to the potential diverse roles of *CASP9*, and gene levels could be indicative of CASP9 function and how this relates to the manifestation of pain. In contrast, as discussed above, *PLAC8* was exclusively significant in the low S-LANSS score group (indicative of nociceptive pain) and may help contribute to identifying robust quantitative biomarkers in unravelling mixed pain types with varying neuropathic/nociceptive components. It is important to note that the S-LANSS questionnaire does not provide a definitive diagnosis but is commonly used to inform diagnosis. Within a subset of the ‘neuropathic pain’ group (clinically referred at recruitment), there appears to be a group with mixed pain or pain not of clear neuropathic origin, depending on the clinical assessment. It is important to distinguish neuropathic pain from nociceptive pain as they require different courses of treatment. However, limitations of the S-LANSS method and similar questionnaires are the reason objective diagnostic tests, which use biomarkers, are necessary.

It must be noted that whilst these genes can provide insights into disease processes, they may not necessarily be useful as a biomarker in isolation as they may relate to other diseases or complex clinical profiles. One of the secondary aims of this study was to identify a set of biomarkers that may be used in combination for the diagnosis and monitoring of neuropathic pain. This was performed as a preliminary test into the usefulness of biomarker combinations in predicting pain type, as it is likely that a practical clinical test for neuropathic pain will include an array of biomarkers. ROC analyses reveal that the single-gene combination (*PLAC8*, *ROMO1*, *A3GALT2*) achieved the highest level of discriminatory power (AUROC = 0.919) and may be useful as a test to discriminate pain type amongst patients presented as having either neuropathic or nociceptive pain (LR = 7.62) (Table 6; Fig. 3). Several gene combinations reveal high discriminatory ability in the comparison of neuropathic patients versus controls, with the highest level of discriminatory power achieved by the combination (*SH3BGRL3*, *TMEM88*, *CASP9*). The *CASP9* gene was featured in all of the five combinations selected, yielding the highest levels of discriminatory power (Table 7; Fig. 3). All the above gene combinations revealed higher levels of discriminatory power and test utility in comparisons of neuropathic pain patients versus control patients. However, the comparison of neuropathic pain patients versus nociceptive pain patients (*PLAC8*, *ROMO1*, *A3GALT2*) may have more clinical significance in the context of delineating mixed pain types, including nociceptive vs neuropathic pain.

**Fig. 4:**
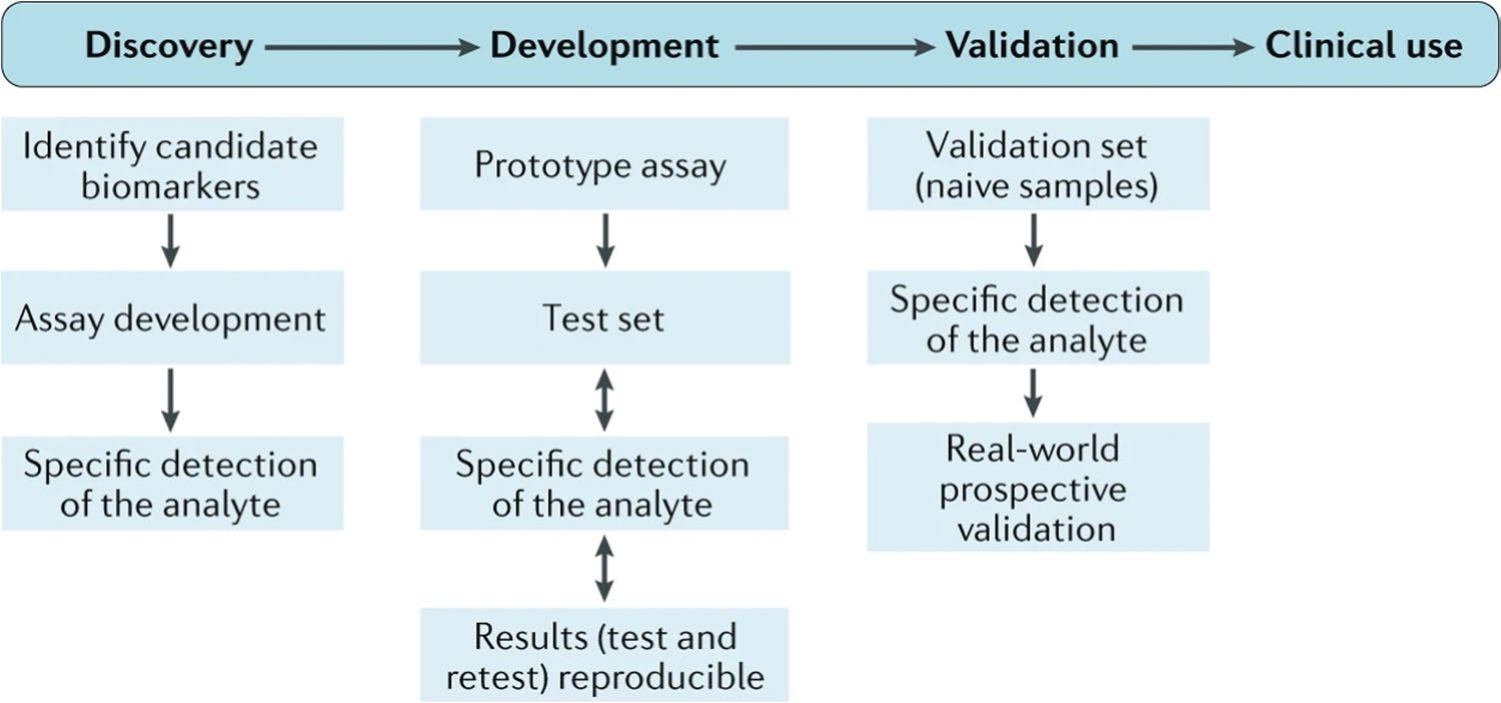
Steps to identify and develop biomarkers for clinical use. The first step in biomarker discovery includes biomarker identification and assay development. Biomarkers can either be identified in preclinical models and reverse-translated to humans or vice versa. The next step is biomarker development, which includes determining that the biomarker or analyte is easily measurable, and that the detection method is reliable and reproducible. A reproducible, reliable, sensitive, and specific biomarker or combination of biomarkers will then be in a position for clinical use. The figure has been adopted from Davis et al. (2020) [59] and permissions have been acquired from the Springer Nature Copyright Clearance Centre

Herein, we have identified a series of molecules that have been reverse-validated from rat DH to human blood, as well as additional molecules identified bioinformatically. The results presented have identified potential biomarkers, and that blood biomarker combinations could be useful for predicting pain type in patients. We also perceive that in combination with the patient measures, these reverse-translated gene expression profiles could function as robust composite multidimensional biomarkers [58], and a more complex mathematical and/or artificial intelligence approach could enhance the ability to stratify patients more effectively. We will now look to develop these biomarkers further for clinical use. This will require us to further validate our profiles in a prospective or validation set (Fig. 4). Figure 4 shows some of the key steps in biomarker development for clinical use [59], and our research has clearly identified and developed a set of biomarker profiles from preclinical to clinical analysis that can now be validated prospectively for potential clinical use.

## Supplementary Information

Below is the link to the electronic supplementary material.

Supplementary file1 (DOCX 17 KB)

## Data Availability

The datasets generated and/or analysed during the current study are not publicly available, but available from Dr McHugh on reasonable request.
