# Supplementary material for: The Identification of Human Translational Biomarkers of Neuropathic Pain and Cross-Species Validation Using an Animal Model
Source: Mol Neurobiol. 2022 Nov 24;60(3):1179–94. doi: 10.1007/s12035-022-03124-7 (PMC9899164; doi:10.1007/s12035-022-03124-7)
Supplement: Supplementary file 1 — Supplementary file1 (DOCX 17 KB) [file 12035_2022_3124_MOESM1_ESM.docx]

**Supplementary Table 1: Gene primer sequences used for human qRT-PCR analysis**

| Gene | Forward Primer | Reverse Primer |
| --- | --- | --- |
| *A3GALT2* | CCTCACCATTGGGCTGACTA | CGTAGTACATCACGCTCTGGC |
| *ANXA1* | GCCTTGTATGAAGCAGGAGAAAG | CACTCTGCGAAGTTGTGGATAGC |
| *CASP1* | GGCATGACAATGCTGCTACA | CCAGCTCTGTAGTCATGTCCG |
| *CASP3* | GCGTGTCATAAAATACCAGTGGA | CCATCCTTTGAATTTCGCCAAG |
| *CASP4* | GGAGCTACTTGAGGGTCTGGA | GTGCTCTGGTCTGGTAGCAA |
| *CASP5* | ACTTTGTCCTCGTGAAGAATTCC | CGTCTGCGGTCCTCTCTC |
| *CASP8* | GGGAACTTCAGACACCAGG | CTCAATTCTGATCTGCTCACTTC |
| *CASP9* | TGGACATTGGTTCTGGAGGA | CCATGCTCAGGATGTAAGCC |
| *CCR5* | TGTCCTTCTCCTGAACACCT | GCGTCATCCCAAGAGTCTCT |
| *CD4* | GGATAGTGGCACCTGGACAT | CCTTCTGGAAAGCTAGCACCA |
| *CYCS* | GGAGGCAAGCACAAGACTGG | CGGCTGTGTAAGAGTATCCAGG |
| *FPR2* | GCTGCATTTGTGTCCTGCAT | ATCCAAGGTCCGACGATCAC |
| *NLRP3* | GACCAGCCAGAGTGGAATGA | GACAACTGCAACCTCACGTC |
| *OMP* | CTCACCAACCTCATGACACG | CGTCGGCCTCATTCCAATCT |
| *PLAC8* | TCCCTGGATCTATTTGTGATGAC | AAGTACGCATGGCTCTCCTT |
| *ROMO1* | ACCGTGTCAAAATGGGCTTC | CGCATTCCGATCCTGAGACAG |
| *SH3BGRL3* | CTCCCGCGAAATCAAGTCC | CCAAGGCTCGCATCTCATC |
| *TMEM88* | GCTTCCTCTGCCACTCTCA | CAGGAATCCGGTGACCAGTA |
| *TXN1* | CTTGGACGCTGCAGGTGATA | GCAACATCATGAAAGAAAGGCTTG |

**Supplementary Table 2: Gene combinations for discriminatory analysis**

| **Gene combination number** | **Constituent genes** |
| --- | --- |
| 1 | *A3GALT2, SH3BGRL3, TMEM88* |
| 2 | *CASP1, CASP4, CASP5* |
| 3 | *FPR2, SH3BGRL3, TMEM88* |
| 4 | *FPR2, CCR5, CD4* |
| 5 | *FPR2, ANXA1, PLAC8* |
| 6 | *SH3BRL3, TMEM88, PLAC8* |
| 7 | *CASP4, CASP5, CASP9* |
| 8 | *CASP4, CASP5, CASP8* |
| 9 | *CASP5, CASP8, CASP9* |
| 10 | *CASP9, CASP5, TMEM88* |
| 11 | *CASP8, CASP5, TMEM88* |
| 12 | *CASP9, CASP5, FPR2* |
| 13 | *CASP8, CASP5, FPR2* |
| 14 | *TMEM88, FPR2, CASP8* |
| 15 | *TMEM88, FPR2, CASP9* |
| 16 | *TMEM88, FPR2, CASP5* |
| 17 | *PLAC8, ROMO1, A3GALT2* |
| 18 | *CASP5, CCR5, SH3BGRL3* |
| 19 | *CASP4, CCR5, SH3BGRL3* |
| 20 | *CASP4, CASP5, CCR5* |
| 21 | *CASP4, CASP5, SH3BGRL3* |
| 22 | *SH3BGRL3, TMEM88, CASP5* |
| 23 | *SH3BGRL3, TMEM88, CASP9* |
